# Juvenile idiopathic arthritis presenting with orbital pseudotumor: A rare pediatric association

**DOI:** 10.1016/j.radcr.2026.06.028

**Published:** 2026-07-04

**Authors:** Kawtar EL Jebbouri, Chaimaa Jabbari, Ali Haidar, Lina Belkouchi, Siham El Haddad, Nazik Allali, Latifa Chat

**Affiliations:** Children’s hospital, Ibn Sina University Hospital Center, Rabat, Morocco

**Keywords:** Inflammatory pseudotumor, Orbit, Juvenile arthritis

## Abstract

Orbital inflammatory pseudotumor is a rare, idiopathic condition characterized by nonspecific inflammation of orbital tissues. Juvenile idiopathic arthritis (JIA), a chronic autoimmune disease, may present with both systemic and extra-articular manifestations, including ocular involvement. This case highlights the importance of a multidisciplinary approach in the diagnosis and management of rare complications of JIA and underscores the need for further investigation into the relationship between systemic autoimmune disorders and localized inflammatory processes.

## Introduction

Idiopathic orbital inflammation, also termed orbital pseudotumor, is a non-granulomatous inflammatory condition of the orbit without an identifiable local or systemic cause. It is the third most common orbital disorder after Graves’ disease and lymphoproliferative lesions [1]. Diagnosis is established by excluding alternative causes through clinical history, physical examination, imaging, and steroid responsiveness. Orbital pseudotumor may involve any orbital structure, including the globe, extraocular muscles, lacrimal glands, and optic nerve. Isolated optic nerve involvement is rare [2]. Histopathological features vary, reflecting differing degrees of inflammation and fibrosis. In children, orbital pseudotumor may occur in association with systemic autoimmune diseases such as JIA.

## Case report

A 12-year-old girl with no significant past medical history presented with a two-year history of asymmetric joint pain affecting the ankles, knees, shoulders, wrists, and metacarpophalangeal joints, associated with myalgia. Laboratory studies showed positive antinuclear antibodies (ANA) and elevated inflammatory markers, including erythrocyte sedimentation rate (ESR) and C-reactive protein (CRP), consistent with a diagnosis of juvenile idiopathic arthritis.

Two years after the onset of articular symptoms, she developed a progressive, painless, bilateral proptosis. Magnetic resonance imaging (MRI) revealed bilateral intraconal pseudotumoral orbital tissue infiltration, isointense on T1-weighted sequences with marked homogeneous enhancement following gadolinium administration (Fig. 1). The lesions were hypointense on T2-weighted sequences, with preserved optic nerve morphology and signal on CISS sequences (Fig. 2). FLAIR sequences showed hypointensity without diffusion restriction (Fig. 3). These findings were consistent with grade III bilateral proptosis, more pronounced on the left, with preserved extraconal fat.

**Fig. 1:**
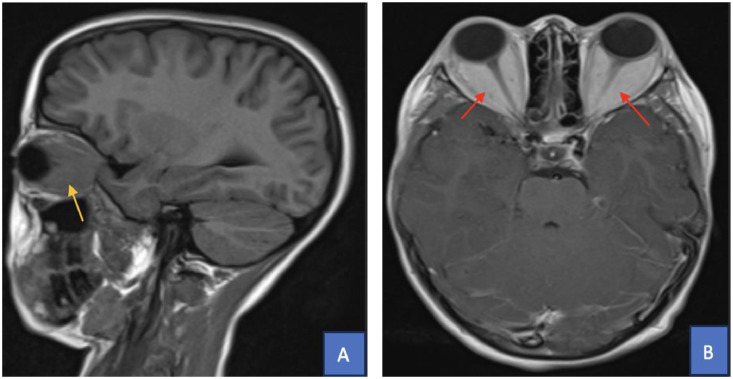
Sagittal T1-weighted imaging (A) and axial T1 post-gadolinium (B) showing bilateral retro-orbital intraconal soft tissue masses with isointense T1 signal (yellow arrow) and homogeneous enhancement after contrast (red arrows).

**Fig. 2:**
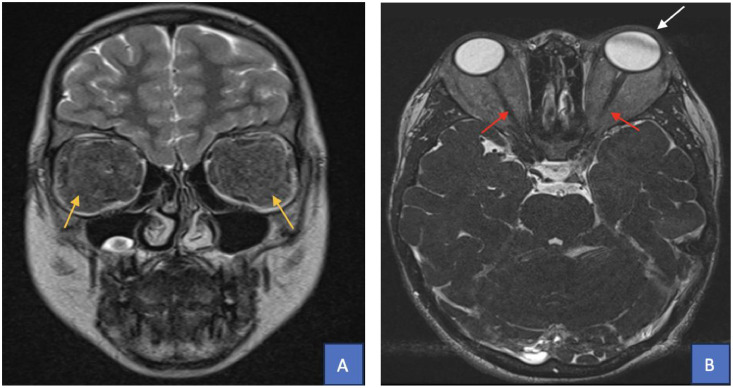
Coronal T2-weighted imaging (A) and CISS sequence (B) showing bilateral retro-orbital intraconal soft tissue masses with T2 hypointensity (yellow arrows), preservation of optic nerve morphology and signal (red arrows), and bilateral grade III proptosis, more marked on the left (white arrow).

**Fig. 3:**
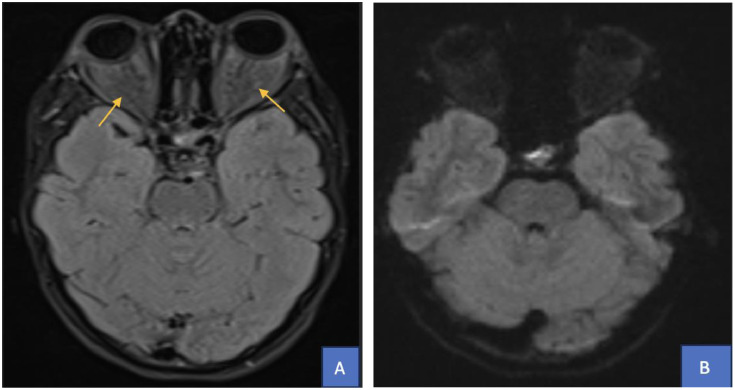
Axial FLAIR (A) and DWI (B) sequences showing bilateral retro-orbital intraconal soft tissue masses with FLAIR hypointensity (yellow arrows) and no restricted diffusion.

Orbital biopsy demonstrated dense lymphoplasmacytic infiltrates interspersed with collagen bundles, confirming a chronic inflammatory process consistent with orbital inflammatory pseudotumor (Fig. 4). The patient was treated with corticosteroids, resulting in significant clinical improvement and complete resolution of ocular symptoms.

**Fig. 4:**
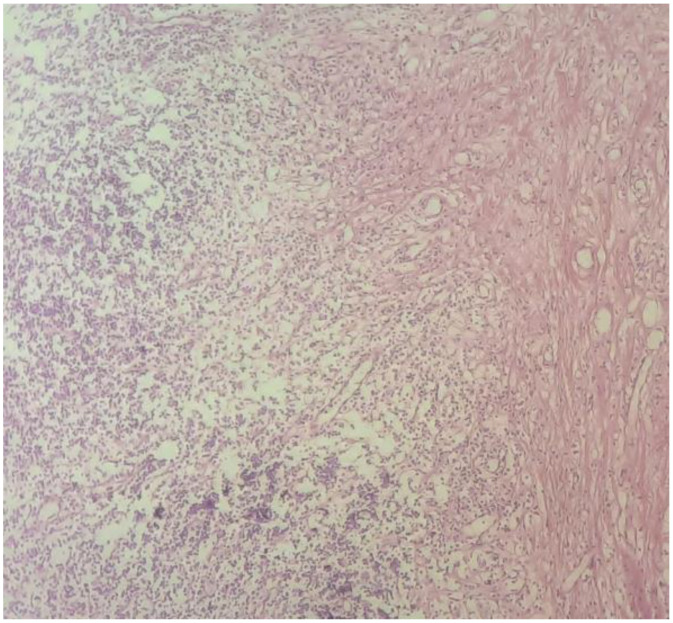
Periorbital biopsy (× 40 magnification) showing infiltration of lymphocytes and plasma cells within fibrous tissue, consistent with orbital inflammatory pseudotumor.

## Discussion

Orbital pseudotumor is a rare clinical and pathological entity characterized by idiopathic inflammation of orbital structures, in the absence of infection, neoplasm, or systemic disease. The term was initially applied broadly to non-neoplastic orbital lesions but is now restricted to idiopathic cases [3]. First described by Birch-Hirschfeld in 1905, its precise etiology remains unclear, although postulated triggers include occult infection, trauma, and chronic immune dysregulation [4]. Accounting for 8–11% of orbital tumors, it is the third most common orbital disorder after thyroid-associated orbitopathy and lymphoproliferative lesions, though it is uncommon in children [5].

The coexistence of orbital pseudotumor and JIA is particularly unusual. Juvenile idiopathic arthritis is an autoimmune condition with well-documented ocular manifestations, most commonly anterior uveitis, but orbital inflammatory disease is rarely reported. Both conditions share an underlying basis of immune dysregulation, with activated T lymphocytes and pro-inflammatory cytokines driving tissue damage. Inflammatory pseudotumor has also been linked to systemic immune-mediated diseases, including rheumatoid arthritis, systemic lupus erythematosus, and IgG4-related disease [6]. These associations raise the possibility of common pathogenic pathways, in which systemic immune activation may predispose to localized orbital inflammation.

Reports in the literature support this link. Weinstein et al. described patients with rheumatoid arthritis who developed orbital pseudotumor, presenting with proptosis and orbital pain and showing rapid improvement with corticosteroids [7]. Cush et al. similarly reported cases associated with adult-onset juvenile rheumatoid arthritis, with diagnosis based on clinical and radiologic findings. In all reports, corticosteroid therapy was effective, emphasizing both the inflammatory nature of the process and the importance of early recognition. To our knowledge, cases of orbital pseudotumor in the pediatric population with JIA remain exceedingly rare, highlighting the novelty of our report.

The diagnosis of orbital pseudotumor is primarily clinical and radiological, supported by rapid response to corticosteroids. MRI findings, including T1 isointensity, T2 hypointensity, and homogeneous post-contrast enhancement, are characteristic and reflect fibrotic and inflammatory changes. Importantly, sparing of tendinous muscle insertions distinguishes the condition from thyroid orbitopathy, while diffusion characteristics help differentiate it from lymphoma [7,8]. In our case, biopsy was performed because of the unusual pediatric presentation and systemic background, which confirmed the diagnosis histologically.

Treatment of orbital pseudotumor typically begins with high-dose systemic corticosteroids, with most patients responding rapidly. However, up to 30% may relapse or develop steroid dependence, necessitating alternative therapies such as radiotherapy, methotrexate, azathioprine, or mycophenolate mofetil [9,10]. Biologic agents targeting TNF-α and interleukin pathways have shown promise in refractory cases, although data remain limited [10]. In children with JIA, the use of immunosuppressants may be guided by systemic disease activity, raising the possibility of shared therapeutic strategies.

## Conclusion

Orbital pseudotumor is a rare manifestation of juvenile idiopathic arthritis, requiring a high index of suspicion in pediatric patients with orbital masses. Recognition of this entity is crucial to avoid unnecessary invasive procedures. Prompt initiation of corticosteroid therapy can achieve rapid and complete resolution. This case emphasizes the importance of considering systemic autoimmune disease in children presenting with orbital pseudotumors.

## Patient consent

Written informed consent for publication was obtained from the patient.
